# Endoscopic ultrasound‐guided fine‐needle biopsy histology with a 22‐gauge Franseen needle and fine‐needle aspiration liquid‐based cytology with a conventional 25‐gauge needle provide comparable diagnostic accuracy in solid pancreatic lesions

**DOI:** 10.1002/jgh3.12642

**Published:** 2021-08-28

**Authors:** Yoichi Tomita, Yuichi Torisu, Masafumi Chiba, Yuji Kinoshita, Takafumi Akasu, Nana Shimamoto, Takahiro Abe, Keisuke Kanazawa, Kazuki Takakura, Shintaro Tsukinaga, Masanori Nakano, Hirobumi Toyoizumi, Masayuki Kato, Masayuki Saruta

**Affiliations:** ^1^ Division of Gastroenterology and Hepatology, Department of Internal Medicine The Jikei University School of Medicine Tokyo Japan; ^2^ Department of Endoscopy The Jikei University School of Medicine Tokyo Japan

**Keywords:** endoscopic ultrasonography guided fine needle aspiration, endoscopic ultrasonography guided fine needle biopsy, Franseen needle, liquid‐based cytology, solid pancreatic lesion

## Abstract

**Background and Aim:**

Fine‐needle biopsy (FNB) needles obtain more core samples and support the shift from cytologic to histologic evaluation; however, recent studies have proposed a superior diagnostic potential for liquid‐based cytology (LBC). This study compared the diagnostic ability of endoscopic ultrasound (EUS)‐guided FNB histology with a 22‐gauge Franseen needle (22G‐FNB‐H) and fine‐needle aspiration (FNA) LBC with a conventional 25‐gauge needle (25G‐FNA‐LBC).

**Methods:**

We analyzed 46 patients who underwent both 22G‐FNB‐H and 25G‐FNA‐LBC in the same lesion during the same endoscopic procedure. This study evaluated the diagnostic ability of each needle, diagnostic concordance between needles, and incremental diagnostic effect of both needles compared to using each needle alone.

**Results:**

The agreement rate for malignancy between both techniques was 93.5% (kappa value = 0.82). There was no significant difference in the diagnostic ability of both methods. 22G‐FNB‐H and 25G‐FNA‐LBC provided an incremental diagnostic accuracy in two (4.3%) cases and one (2.2%) case, respectively.

**Conclusion:**

Our study demonstrated that the diagnostic accuracy of 25G‐FNA‐LBC and 22G‐FNA‐H for solid pancreatic lesions were comparable. A conventional 25‐gauge needle that punctures lesions with ease can be used in difficult cases and according to the skill of the endoscopist.

## Graphical abstract

We analyzed 46 patients who underwent both endoscopic ultrasound‐guided fine‐needle biopsy histology with a 22‐gauge Franseen needle and fine‐needle aspiration liquid‐based cytology with a conventional 25‐gauge needle in the same lesion during the same endoscopic procedure. There was no significant difference in the diagnostic ability of both methods. Our study suggests that a conventional 25‐gauge needle that punctures lesions with ease can be used in difficult cases and according to the skill of the endoscopist.

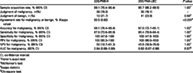

## Introduction

The first endoscopic ultrasonography (EUS)‐guided fine‐needle aspiration (FNA) biopsy was performed by Villman *et al*. in 1992.^1^ EUS‐FNA has been regarded as an effective diagnostic technique for pancreatic lesions, and three meta‐analyses have demonstrated sensitivity and specificity levels of 85–89% and 96–99%, respectively.^2^, ^3^, ^4^ Pathologic evaluation conventionally utilized cytologic findings; however, this was difficult to perform on small samples without core tissue. In comparison, histopathologic evaluation provides more information on the histologic and immunohistochemical structure of solid pancreatic lesions, which facilitate more accurate diagnoses.^5^

Several needles are available for EUS‐FNA and differ based on the size, tip shape, and material of the puncture needle and sheath material; however, there is no clear superior option. In general, needles with larger bores collect more specimens but are stiffer, especially when used with an angled scope in the duodenum, which increases the difficulty of the procedure.^6^, ^7^, ^8^ As such, multiple studies have demonstrated that among technically successful procedures, 19‐ or 22‐gauge needles provide more accurate histologic diagnoses compared to 25‐gauge needles. However, when all procedures, including technical failures, were considered, the accuracy of histologic diagnosis was not influenced by needle size.^6^

Fine‐needle biopsy (FNB) needles were recently introduced for core tissue sampling.^9^ Third FNB needles have four tip designs and it includes a forward‐bevel needle (20G ProCore, Cook Medical, Bloomington, Indiana, USA), fork‐tip needle (SharkCore, Medtronic, Minneapolis, Minneapolis, USA), and two types of Franseen‐type needles (Acquire, Boston Scientific, Marlborough, Massachusets, USA; (TopGain, Mediglobe, Achenmühle, Germany). Among these needles, the Acquire Franseen needle (Boston Scientific), 22‐gauge caliber needle with three symmetric heels at the tip (Franseen geometry) has been extensively studied for core tissue sampling in histopathologic assessment.^10^, ^11^, ^12^, ^13^, ^14^, ^15^, ^16^

Previous studies have also proposed that 25G‐FNA needles were superior to 22G‐FNA needles in terms of the overall diagnostic accuracy of cytologic findings.^6^, ^17^, ^18^ While cytologic evaluation has traditionally been performed with conventional smear cytology, whereas recent studies have proposed that precipitation‐based and liquid‐based cytology (LBC) (SurePath, Becton, Dickinson and Company, Franklin Lakes, New Jersey, USA) provides superior diagnostic detail in the absence of rapid on‐site evaluation (ROSE).^19^ We compared the diagnostic ability of EUS‐FNB histological examination using 22G‐FNB‐H and EUS‐FNA‐ liquid‐based cytological examination using a conventional 25G‐FNA‐LBC in solid pancreatic lesions.

## Methods

### Study design and population

This prospective study was approved by the Ethics Committee of The Jikei University School of Medicine for Biomedical Research 30–436 (9457), and all patients provided informed consent for EUS‐guided tissue sampling.

This was a single‐center, retrospective study. Data of consecutive patients who underwent EUS‐FNB or EUS‐FNA of solid pancreatic lesions at the Jikei University Hospital between October 2018 and September 2020 were collected from the medical database and analyzed.

A total of 110 patients underwent EUS‐FNB or EUS‐FNA with different needles. We analyzed the data of 46 patients who underwent both EUS‐FNB with the Acquire 22‐gauge Franseen needle (Boston Scientific) and EUS‐FNA with the EZ Shot 3 plus 25‐gauge end‐cut type needle (Olympus Optical, Tokyo, Japan) for the same lesion during the same endoscopic procedure.

### Surgical technique

All EUS‐FNB and EUS‐FNA procedures were performed using a curved linear EUS (GF‐UCT260; Olympus Medical Systems, Tokyo, Japan) with color Doppler function (EU‐ME2 Premier Plus; Olympus Medical Systems). Patients were placed under moderate sedation using intravenous pethidine hydrochloride and midazolam. The lesion and regional and collateral vasculature were visualized using EUS with a color Doppler function, and the lesion was punctured using the appropriate needle. Using two types of needles was decided by the attending physician before the first puncture was performed. Approximately 20 to‐and‐fro movements of the needle were performed within the lesion, and the stylet was slowly withdrawn with a fanning motion until the needle was completely removed. The material obtained by EUS‐FNB was transferred into a specimen bottle with formalin for histologic examination, whereas the material obtained by EUS‐FNA was transferred into a SurePath (Becton, Dickinson, and Company) bottle for cytologic examination.

At our hospital, rapid on‐site cytologic evaluation (ROSE) was not performed. The number of passes was decided at the discretion of the attending physician; however, EUS‐FNB or EUS‐FNA was repeated until adequate visible core tissue was obtained macroscopically. All procedures were performed by two experts (≥5 years of EUS‐FNA experience) or by six trainees (<5 years of EUS‐FNA experience) under the supervision of the experts. EUS‐FNB and EUS‐FNA have performed alternately for the same lesions.

### Final diagnosis

The final diagnosis was determined by the pathologic diagnosis of a surgically resected specimen. EUS‐FNB or EUS‐FNA diagnosis of a malignant tumor that was compatible with clinical outcomes, or EUS‐FNB or EUS‐FNA diagnosis of a benign tumor that did not demonstrate clinical deterioration for at least six months.

### Evaluation

This study evaluated the sample acquisition rate and diagnostic ability of each method, diagnostic concordance between 22G‐FNB‐H and 25G‐FNA‐LBC, and incremental effect of using both needles compared to using each needle alone. Acquisition rate and diagnostic ability were evaluated for each session instead of each needle pass. The adequacy of the samples was determined by the pathologist and was defined as either satisfactory or unsatisfactory to provide a definitive pathologic diagnosis. Diagnostic performance was calculated based on the ability to discriminate between benign and malignant lesions. Non‐diagnostic samples were considered benign, and suspected malignant samples were considered malignant. Diagnostic accuracy was defined as the sum of true‐positive and true‐negative results divided by the total number of lesions.

We also investigated the incidence of procedural complications per patient. Information regarding immediate and delayed adverse events was obtained by reviewing operative technique reports and emergency room visits or hospitalizations records, respectively.

### Statistical analysis

Continuous variables were presented as mean ± standard deviation (SD), and categorical variables are presented as numbers and percentages. Statistical comparison of categorical variables was performed using the chi‐square test, Fisher's exact test, or McNemer's test. Inter‐rater agreement was assessed using kappa statistics. A *P*‐value <0.05 in a two‐tailed test was considered statistically significant. All statistical analyses were performed using Stata version 15 (StataCorp LP, College Station, Texas, USA).

## Results

### Patient and procedure characteristics

The patient and procedure characteristics are shown in Table 1. The patients included 31 men and 15 women with a mean age of 66.8 ± 10.3 years. The mean largest lesion diameter was 28.7 ± 10.6 mm. Eighteen (39.1%) and 28 (60.9%) lesions were located in the head or body and tail of the pancreas, respectively. The final diagnoses included 41 (89.1%) malignant and five (10.9%) benign lesions. The malignant lesions included 33 pancreatic ductal cancers, three intraductal papillary mucinous carcinomas, and two neuroendocrine tumors. One diagnosis of acinar cell carcinoma, malignant lymphoma, and metastasis of renal cell carcinoma was also documented. The benign lesions included four cases of autoimmune pancreatitis and one case of chronic pancreatitis. The mean number of needle passes was 2 ± 0.5 for both EUS‐FNB and EUS‐FNA. In the present study, no procedural adverse events were observed.

**Table 1:** Patient and procedure characteristics is paired between 22G‐FNB‐H and 25G‐FNA‐LBC

	Total (*n* = 46)
Age, mean ± SD	66.8 ± 10.3
No. men, *n* (%)	31 (67.4)
Lesion size, mm mean ± SD	28.7 ± 10.6
Pancreatic location, *n* (%)	
Head	18 (39.1)
Body/Tail	28 (60.9)
Malignant disease, *n* (%)	41
Pancreatic ductal adenocarcinoma	33 (71.7)
Intraductal papillary mucinous carcinoma	3 (6.5)
Neuroendocrine tumor	2 (4.4)
Acinar cell carcinoma	1 (2.2)
Malignant Lymphoma	1 (2.2)
Metastasis of renal cell carcinoma	1 (2.2)
Benign disease, *n* (%)	5
Autoimmune pancreatitis	4 (8.7)
Chronic Pancreatitis	1 (2.2)
No. passes, mean ± SD	4.0 ± 0.8
22G‐FNB‐H	2.0 ± 0.5
25G‐FNA‐LBC	2.0 ± 0.5
Complications (EUS‐FNB and FNA), *n* (%)	0 (0)

EUS‐FNA, Endoscopic Ultrasound‐Fine Needle Aspiration.

### Outcomes

**Figure 1:**
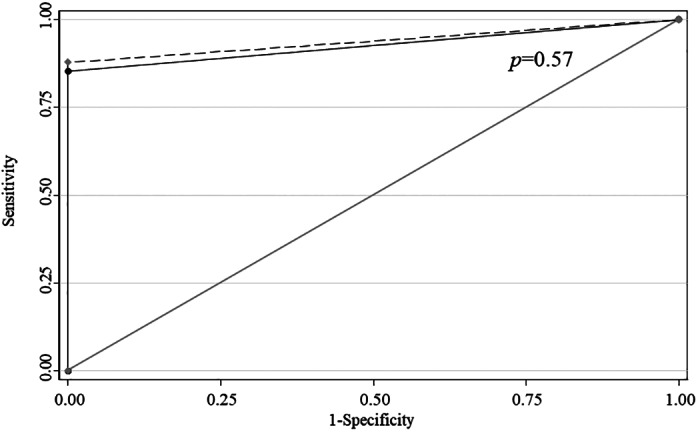
Receiver characteristic operating curves comparing the diagnostic accuracy of 25‐gauge fine‐needle aspiration cytology (25G‐FNA‐C) *versus* 22‐gauge fine‐needle aspiration biopsy histology (22G‐FNB‐H) the area‐under‐the‐curves for diagnosing malignancy with 25G‐FNA‐C and 22G‐FNB‐H is 0.93 and 0.94, respectively. There is no significant difference between the two methods. 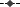
22G‐FNB‐H AUC = 0.94; 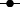
25G‐FNA‐LBC AUC = 0.93; 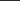
Reference

The diagnostic performance of 22G‐FNB‐H and 25G‐FNA‐LBC are shown in Table 2 and Figure 1, respectively. The sample acquisition rates were 89.1% and 95.7% for 22G‐FNB‐H and 25G‐FNA‐LBC, respectively; there was no significant difference in the acquisition rate between both methods.

**Table 2:** Diagnostic performance between 22G‐FNB‐H and 25G‐FNA‐LBC

	22G‐FNB‐H	25G‐FNA‐LBC	*P* value
Sample acquisition rate, % (95% CI)	89.1 (76.4–96.4)	95.7 (85.2–99.5)	1.00^†^
Judgment of malignancy, *n* (%)	36 (78.3)	35 (76.1)	–
Judgment of benign, *n* (%)	10 (21.7)	11 (23.9)	0.56^‡^
Agreement rate for malignancy or benign, % (Kappa value)	93.5 (0.82)		<0.001^§^
Accuracy for malignancy, % (95% CI)	89.1 (76.4–96.4)	87.0 (73.7–95.1)	1.00^†^
Sensitivity for malignancy, % (95% CI)	87.8 (73.8–95.9)	85.4 (70.8–94.4)	1.00^†^
Specificity for malignancy, % (95% CI)	100 (47.8–100)	100 (47.8–100)	1.00^†^
PPV for malignancy, % (95% CI)	100 (90.3–100)	100 (90.0–100)	1.00^†^
NPV for malignancy, % (95% CI)	50 (18.7–81.3)	45.5 (16.8–76.6)	1.00^†^
AUC for malignancy, (95% CI)	0.94 (0.89–0.99)	0.93 (0.87–0.98)	0.57^¶^

CI, confidence interval.

^†^ Fisher's exact test.

^‡^ McNemer's test.

^§^ kappa statistic.

^¶^ Chi‐square test.

22G‐FNB‐H identified 36 (78.3%) malignant and 10 (21.7%) benign lesions, whereas and 25G‐FNA‐LBC identified 35 (76.1%) malignant and 11 (23.9%) benign lesions. The agreement rate between 22G‐FNB‐H and 25G‐FNA‐LBC was 93.5% (kappa value = 0.82).

The accuracy, sensitivity, specificity, positive predictive value, negative predictive value, and area‐under‐the‐curve for diagnosing malignancy were 89.9%, 87.8%, 100%, 100%, 50%, and 0.94, respectively, for 22G‐FNB‐H and 87.0%, 85.4%, 100%, 100%, 45.5%, and 0.93, respectively, for 25G‐FNA‐LBC. There was no significant difference in the diagnostic ability between both methods.

22G‐FNB‐H provided an incremental diagnostic accuracy in two (4.3%) cases of adenocarcinoma, whereas 25G‐FNA‐LBC provided an incremental diagnostic accuracy in one (2.2%) case of adenocarcinoma. Both 22G‐FNB‐H and 25G‐FNA‐LBC missed four cases of malignancy, which were diagnosed with surgical resection or clinical follow‐up procedure. These cases included pancreatic ductal adenocarcinoma (*n* = 1) and intraductal papillary mucinous carcinoma (*n* = 3).

## Discussion

Our results suggested that the diagnostic accuracy of FNA‐LBC with a conventional 25‐gauge needle and FNB histology with a 22‐gauge Franseen needle were comparable. Three meta‐analyses on solid pancreatic lesions demonstrated that FNB needles provide better diagnostic accuracy and require fewer needle passes than FNA needles.^20^, ^21^, ^22^ FNB with a 22‐gauge Franseen needle has also been shown to provide better tissue quality and more adequate nucleic acid yield for microsatellite instability evaluation compared to FNA with a conventional 22‐gauge needle.^13^, ^14^ As such, the 22‐gauge Franseen needle holds strong promise for precision diagnostics; however, its three symmetrical heel tip design increases the puncture resistance, making it more difficult to use. In comparison, the conventional 25‐gauge end‐cut type needle has a low puncture resistance and is highly flexible. The EZ Shot 3 plus 25‐gauge needle (Olympus Optical) used in this study has a multi‐layer coil sheath and Mengini tip, which provide excellent flexibility and puncturing ability. This needle is easy to use from a technical perspective and punctures the lesion exceptionally well. However, EUS‐FNA with a conventional 25‐gauge needle collects only a small amount of sample, which increases the risk for an inadequate yield for histologic diagnosis.^6^ Smaller samples are more suited for cytologic examination. Conducting a cytologic assessment on samples acquired through FNA with a conventional 25‐gauge needle provides higher diagnostic accuracy than a histologic assessment.^6^, ^17^, ^18^ Compared to CS cytology without ROSE, the precipitation‐based LBC technique (SurePath; Becton, Dickinson and Company) has attracted attention because of its sensitivity for malignant pancreatic lesions.^19^ We performed precipitation‐based LBC for samples acquired through FNA with a conventional 25‐gauge needle to increase the diagnostic accuracy of our technique.

Our data demonstrated that 22G‐FNB‐H and 25G‐FNA‐LBC had similar diagnostic accuracy rates. Moreover, our data showed that the agreement rate between both groups was 93.5% (kappa value = 0.82), which suggested that the needle size was not a significant factor for accurate diagnosis. Only three (6.5%) diagnoses differed between the two groups, and there was no significant difference in the incremental diagnostic accuracy when both 22G‐FNB‐H and 25G‐FNA‐LBC were performed in the same lesion. Our data suggested that there was a limited diagnostic benefit to performing both procedures in the same lesion, and the combined use of both needles increased the overall cost.

Histologic evaluation of EUS‐FNB core tissue samples often provides more specific diagnoses because the samples can be evaluated for tissue architecture and immunostaining. However, a malignant diagnosis by cytology is enough in clinical practice, and additional tests are rarely performed.

The results of this study suggest that conventional 25‐gauge needles should be used in difficult cases and according to the skill of the endoscopist because these needles are relatively easy to use.

During this study period, 16 patients underwent EUS‐FNA‐LBC with the EZ Shot 3 plus 25‐gauge needle (Olympus Medical) alone because the lesions in these patients were considered difficult to puncture. The diagnostic accuracy in these cases was 87.5%, which was similar to that published for such cases (data not shown).

This study has several limitations. First, the total number of enrolled patients was small, and we retrospectively collected data from a single center. A prospective study with a larger number of cases from multiple hospitals may provide better superior data. Second, our study may have been prone to selection bias because we only analyze lesions that could be punctured with both types of needles. Third, we did not examine whether the order in which the needles were used contributed to the final outcomes; it is possible that the first needle affected the quality of the sample obtained with the second needle. Fourth, both trainees and experts performed the procedures in our study, and the quality of the sample may be affected by the skill of the performer. Lastly, we evaluated the diagnostic ability of each needle in each session because we could not compare the diagnostic ability of each pass.

### Conclusions

Our study demonstrated that the diagnostic accuracy of 25G‐FNA‐LBC and 22G‐FNA‐H for solid pancreatic lesions were comparable. Conventional 25‐gauge needles puncture the lesion more easily and should be used in difficult cases and according to the skill of the endoscopist.
